# On using optical maps for genome assembly

**DOI:** 10.1186/gb-2011-12-s1-p41

**Published:** 2011-09-19

**Authors:** Henry Lin, Mihai Pop

**Affiliations:** 1 Center for Bioinformatics and Computational Biology, University of Maryland, College Park, MD 20742, USA

## Background

In this work, we study the benefits of using optical maps to improve genome assembly. Many modern assembly algorithms rely on a de Bruijn graph paradigm to reconstruct a genome from short reads. Ambiguities caused by repeats within the genome cause the final assembly to be broken up into many contigs, because the assembler does not have enough information to find the one correct traversal of the graph. Optical mapping technology can be useful for determining the correct path in the de Bruijn graph, through providing estimates on the locations of one or more restriction enzyme patterns in the genome, thereby constraining the possible traversals of the graph to only those that are consistent with the map. A particular traversal that does not align well with the optical map can be discarded as incorrect. Previous work has shown how to construct optical maps [1,2] for scaffolding contigs [3].

## Methods

Our algorithm relies on a depth-first search strategy. As the depth-first search proceeds and its corresponding sequence is extended, we check whether the resultant sequence would generate an optical map that matches the optical map of the genome. If the candidate *in silico* optical map matches the optical map of the genome, we proceed with the depth-first search. Otherwise, we backtrack in the depth-first search until we find a path that covers the entire graph and whose sequence has an optical map that matches the optical map of the entire genome. Although the total number of paths in the de Bruijn graph can be exponential in the number of nodes and edges in the graph [4], a reference optical map can effectively prune the search space of paths. To improve performance, we start by finding edges in the de Bruijn graph that can be uniquely placed on the optical map. These edges, which we call landmark edges, can also help guide our depth-first search. Although there may be multiple paths in the de Bruijn graph that can yield sequences with optical maps that match the genome’s optical map, these paths all yield very similar sequences in most cases.

## Results

Given modest assumptions about the errors in the optical map, initial simulations show that our algorithm is very effective at assembling bacterial genomes, given read lengths of 100 or longer. The majority of our assemblies match the original sequences used in our simulations very closely. We will also present the results of simulations aimed at measuring the effect of errors on the correctness of the reconstruction and at measuring how the choice of restriction enzymes can improve the sequence assembly.

## Conclusions

Our work shows that optical maps can be used effectively to aid in genome assembly. We are currently extending our approach to handle much larger graphs and to tolerate higher amounts of mapping error. In our final assembly, we would also like to be able to detect and mark regions that we are less certain about and regions that we are confident are correct.

## References

[B1] Aston C Mishra B Schwartz DC Optical mapping and its potential for large-scale sequencing projects Trends Biotechnol 1999 17 297 302 10.1016/S0167-7799(99)01326-8 10370237

[B2] Valouev A Schwartz DC Zhou S Waterman MS An algorithm for assembly of ordered restriction maps from single DNA molecules Proc Natl Acad Sci USA 2006 103 15770 15775 10.1073/pnas.0604040103 17043225 PMC1635078

[B3] Nagarajan N Read TD Pop M Scaffolding and validation of bacterial genome assemblies using optical restriction maps Bioinformatics 2008 24 1229 1235 10.1093/bioinformatics/btn102 18356192 PMC2373919

[B4] Kingsford C Schatz MC Pop M Assembly complexity of prokaryotic genomes using short reads BMC Bioinformatics 2010 11 21 10.1186/1471-2105-11-21 20064276 PMC2821320

